# Adenanthin, a Natural *ent*-Kaurane Diterpenoid Isolated from the Herb *Isodon adenantha* Inhibits Adipogenesis and the Development of Obesity by Regulation of ROS

**DOI:** 10.3390/molecules24010158

**Published:** 2019-01-03

**Authors:** Jing Hu, Xingren Li, Weifeng Tian, Yanting Lu, Yuhui Xu, Fang Wang, Wanying Qin, Xiuli Ma, Pema-Tenzin Puno, Wenyong Xiong

**Affiliations:** 1 State Key Laboratory of Phytochemistry and Plant Resources in West China, Kunming Institute of Botany, Chinese Academy of Sciences, Kunming 650201, China; hujing@mail.kib.ac.cn (J.H.); lixingren@mail.kib.ac.cn (X.L.); tianweifeng@mail.kiz.ac.cn (W.T.); luyanting@mail.kib.ac.cn (Y.L.); xuyuhui@mail.kib.ac.cn (Y.X.); wangfang@mail.kib.ac.cn (F.W.); qinwanying@mail.kib.ac.cn (W.Q.); maxiuli@mail.kib.ac.cn (X.M.); 2 University of the Chinese Academy of Sciences, Beijing 100049, China; 3 Yunnan Key Laboratory of Natural Medicinal Chemistry, Kunming 650201, China

**Keywords:** adenanthin, adipogenesis, reactive oxygen species, obesity, 3T3-L1

## Abstract

Adenanthin, a natural *ent*-kaurane diterpenoid extracted from the herb *Isodon adenantha*, has been reported to increase intracellular reactive oxygen species in leukemic and hepatocellular carcinoma cells. However, the function and mechanism of the compound in adipogenesis and the development of obesity is still unknown. In this study, we demonstrated that adenanthin inhibited adipogenesis of 3T3-L1 and mouse embryonic fibroblasts, and the underlying mechanism included two processes: a delayed mitotic clonal expansion via G0/G1 cell cycle arrest by inhibiting the RB-E2F1 signaling pathway and a reduced C/EBPβ signaling by inhibiting the expression and activity of C/EBPβ during mitotic clonal expansion. Furthermore, adenanthin significantly reduced the growing body weight and adipose tissue mass during high-fat diet-inducing obesity of mice, indicating the beneficial effects of adenanthin as a potential agent for prevention of obesity.

## 1. Introduction

Nowadays the prevalence of obesity has become a serious worldwide health problem [1], because it is closely correlated to the risk of various diseases, such as cardiovascular disease, type 2 diabetes, fatty liver disease, certain types of cancer, asthma, osteoarthritis, and premature death [2,3]. An energy imbalance between energy intake and energy expenditure is the fundamental cause of obesity [4]. When energy intake is greater than energy expenditure, it leads to overweight or obesity. Excess energy is mainly stored in adipose tissue in the form of triacylglycerols which leads adipocytes to alter in cell number (hyperplasia) and/or cell size (hypertrophy) [5]. Lately, the anti-obesity property of various natural products has attracted more and more attention. For example, carnosic acid, 6α-Hydroxylup-20(29)-en-3-on-28-oic acid, andrographolide, curcumin and kudinoside-d have been indicated to be effective for inhibiting adipocyte differentiation and lipid accumulation [6,7,8,9,10].

Adipogenesis is a process of cell differentiation from preadipocytes to mature adipocytes. The murine 3T3-L1 cells and mouse embryonic fibroblasts (MEFs), in vitro models, have proved useful for studying this process [11,12]. Adipogenesis can be divided into several phases including growth arrest, mitotic clonal expansion (MCE), lipid accumulation, and late phase of differentiation [13,14]. Particularly, MCE is a necessary stage for terminal differentiation where growth-arrested cells re-enter the cell cycle progress synchronously, resulting in an increase in cell numbers [15,16,17]. In general, the transition from growth arrest into the S phase depends on the reactivation of the G1 cyclins/cdks and the retinoblastoma protein RB-E2F pathway which controls the G1/S transition of the cell cycle [18]. Meanwhile, C/EBPβ expression is rapidly activated after adipogenesis-inducing cocktail during the early phase of adipocyte differentiation, which is a prerequisite for successful differentiation [19]. However, until the growth-arrested cells synchronously transit across the G1 to the S phase during MCE, C/EBPβ acquires the ability to bind to DNA [20]. After C/EBPβ binds to DNA, the expression of mainly later regulators of adipogenesis, including C/EBPα and PPARγ, are transcriptionally activated [17]. 

Reactive oxygen species (ROS) generation was observed during adipogenesis two decades ago [21], accumulating research supports that ROS plays an essential role in adipocyte differentiation [22,23]. However, the effect of ROS on adipogenesis is controversial. On the other hand, ROS is shown to inhibit adipocyte differentiation by reducing the DNA-binding activity of C/EBPβ [24] and suppressing MCE [25]. Therefore, evaluating the effect of ROS in adipogenesis is complex, either below or above a certain ROS level adipogenesis fails to progress [26]. 

Adenanthin, an *ent*-kaurane diterpenoid isolated from the herb *Isodon adenantha* [27], has been shown to target Prx 1 and Prx 2 to exert its bioactivity in increasing ROS of leukemic cells [28], inhibiting NF-*κ*B signaling [29] and killing hepatocellular carcinoma cells [30]. However, the effect of adenanthin on adipogenesis and the development of obesity is unknown. In the present study, we investigated the inhibitory effects of adenanthin on adipogenesis of 3T3-L1 and MEFs and HFD-induced development of obesity in mice, and explored the potential molecular mechanisms of ROS underlying these effects.

## 2. Results

### 2.1. Adenanthin Inhibits Adipogenesis in 3T3-L1 Preadipocytes and MEFs

**Figure 1:**
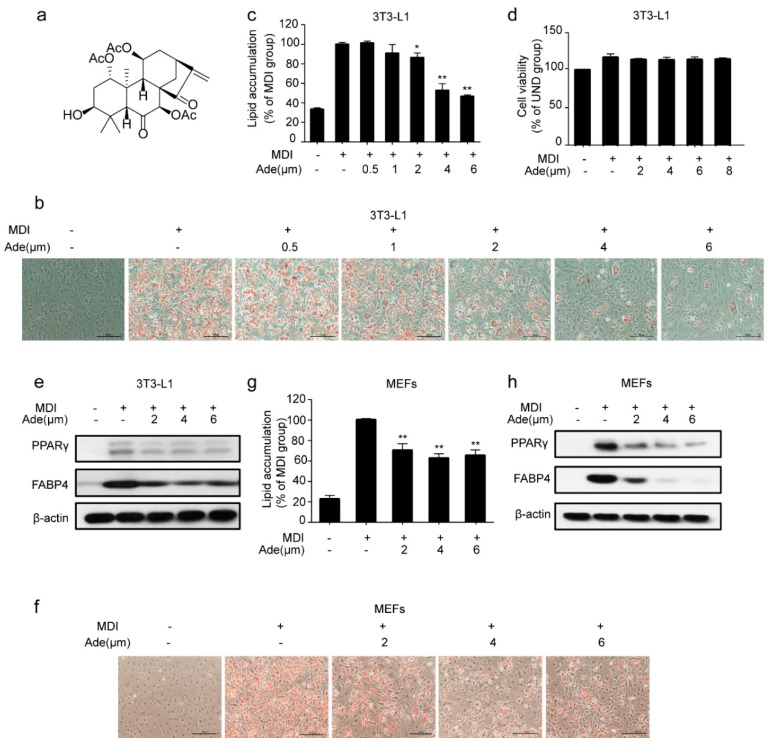
Adenanthin inhibited adipogenesis in 3T3-L1 and mouse embryonic fibroblasts (MEFs). (**a**) Chemical structure of adenanthin. (**b**) Representative images of 3T3-L1 adipocytes treated with MDI, MDI and a series of doses of adenanthin (0.5 to 6 μM) were stained by Oil Red O. Scale bars, 100 μM. (**c**) Quantification of lipid accumulation by measuring Oil Red O intensity of 3T3-L1 cells from (**b**). (**d**) Cell viability of 3T3-L1 adipocytes treated with a series of doses of adenanthin (2 to 8 μM). (**e**) Protein levels of PPARγ and FABP4 of 3T3-L1 adipocytes treated with MDI, MDI and adenanthin (2 to 6 μM). (**f**) Representative images of lipid accumulation of MEFs were visualized by Oil Red O staining after MDI-induced. Scale bars, 100 μM. (**g**) Quantification of lipid accumulation by measuring Oil Red O intensity of MEFs from (**f**). (**h**) Protein levels of PPARγ and FABP4 of MEFs from (**f**). Date are showed as mean ± SD of three independent experiments; * *p* < 0.05, ** *p* < 0.01 versus MDI group.

To evaluate the effect of adenanthin on adipocyte differentiation, we treated 3T3-L1 preadipocytes with a classic adipogenesis-inducing cocktail [31] and measured the level of lipid accumulation by Oil Red O staining at the end of adipogenesis. As shown in Figure 1b, 3T3-L1 pre-adipocytes were induced with MDI cocktail with or without the treatment of adenanthin (0.5, 1, 2, 4, or 6 μM). MDI-stimulated cells were fully differentiated after 7 days, while adenanthin treatments inhibited the differentiation of 3T3-L1. Quantification of Oil Red O showed that treatments with 0.5, 1, 2, 4, and 6 μM adenanthin decreased the lipid content of cells by 0, 9.29%, 13.59%, 47.21%, and 53.49%, respectively (Figure 1c). Particularly, adenanthin showed its strongest inhibition effect at 4 μM, with no effect on cell viability (Figure 1d). Furthermore, we detected the expression of several differentiation markers, including PPARγ and FABP4. As we expected, adenanthin significantly reduced the levels of the two important proteins (Figure 1e).

Moreover, we also attempted to confirm the effect of adenanthin in adipogenesis in MEFs, a type of fibroblast isolated from mouse embryos. Consistent with the above results of 3T3-L1 cells, we observed that adenanthin treatments suppressed lipid accumulation (Figure 1f,g). Similarly, adenanthin treatments also strikingly reduced the levels of PPARγ and FABP4 in MEFs (Figure 1h). 

### 2.2. Adenanthin Functions in the Early Stage of Adipogenesis

**Figure 2:**
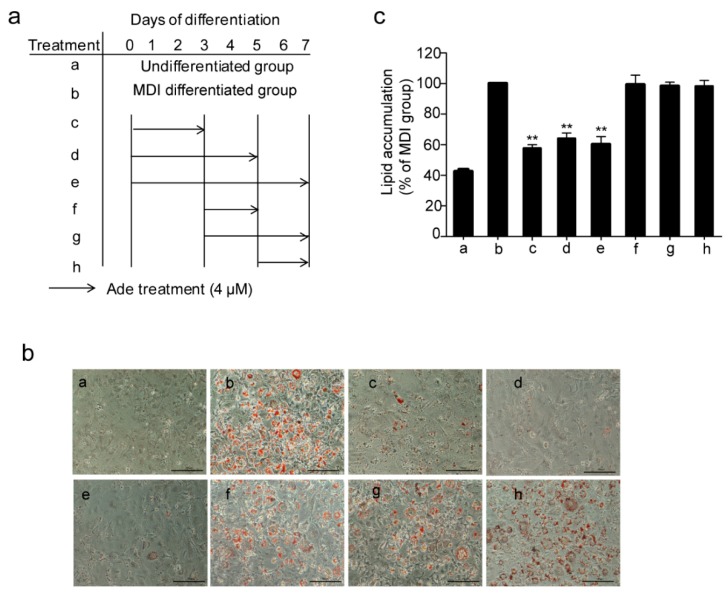
Adenanthin inhibited adipogenesis at the early stages of 3T3-L1 adipocyte differentiation. (**a**) Workflow of multiple treatments of 3T3-L1 during differentiation. (**b**) Representative images of 3T3-L1 adipocytes, treated as (**a**), were stained by Oil Red O. Scale bars, 100 μM. (**c**) Quantification of lipid accumulation by measuring Oil Red O intensity of 3T3-L1 cells from (**b**). Date are showed as mean ± SD of three independent experiments; ** *p* < 0.01 versus MDI group.

Next, we investigated the mechanism of adenanthin on its inhibition of adipogenesis, we treated the 3T3-L1 preadipocytes with adenanthin and MDI medium together at different stages of differentiation as indicated in Figure 2a. As shown in Figure 2b,c, treatments with adenanthin strikingly decreased lipid accumulations by ~42%, ~36%, and ~40% from day 0 to 3, day 0 to 5, and day 0 to 7, whereas treatments on the other days (3–5, 3–7, and 5–7) did not affect lipid accumulation, indicating that adenanthin suppressed adipogenesis by inhibiting the early stage of adipocyte differentiation. 

### 2.3. Adenanthin Suppresses Cell Cycle Progression during MCE

**Figure 3:**
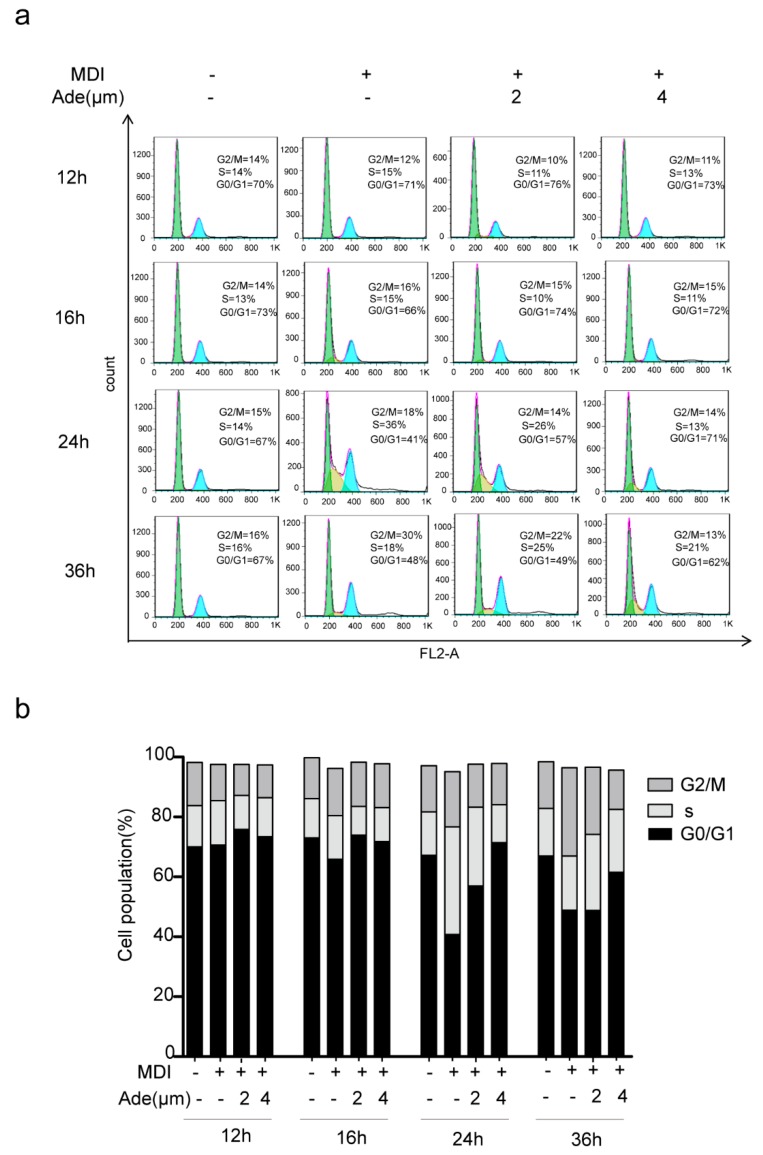
Adenanthin induced a cell cycle arrest during 3T3-L1 adipogenesis. (**a**) Flow cytometry analysis of 3T3-L1 cells treated with MDI, MDI and adenanthin (2, 4 μM) for 12, 16, 24, or 36 h. (**b**) Cell cycle quantification of the cells from (**a**). Date are averages of three independent experiments.

During the process of adipogenesis, the growth-arrested 3T3-L1 preadipocytes go through two sequential rounds of mitosis in the initial 48 h of differentiation which is termed as MCE [16]. Since adenanthin was effective in the early phase of differentiation, we next determined whether adenanthin affected the cell cycle progression during MCE. Therefore, we monitored the changes in DNA content of 3T3-L1 cells using FACS analysis. As shown in Figure 3,b, the data revealed that the undifferentiated cells did not undergo cell cycle progression because most of cells (~70%) resided in G0/G1 phase from 12 to 36 h. The percentage of cells in G0/G1 phase after treatment by MDI alone for 12, 16, 24, or 36 h culture were 70.67%, 65.88%, 40.82%, and 48.91%, respectively. Interestingly, 2 μM MDI and adenanthin-treated cells were gradually shown hailed-tendency in G0/G1 phase as 75.89%, 73.92%, 56.98%, and 48.82% during this period. Moreover, greater adenanthin (4 μM) induced these cells in G0/G1 phase with the values at 73.49%, 71.81%, 71.48%, and 61.51% in the period of culture, demonstrating that adenanthin suppressed cell cycle progression by arresting the cells in G0/G1 phase.

### 2.4. ROS Controls Adenanthin-Mediated Inhibition of MCE

**Figure 4:**
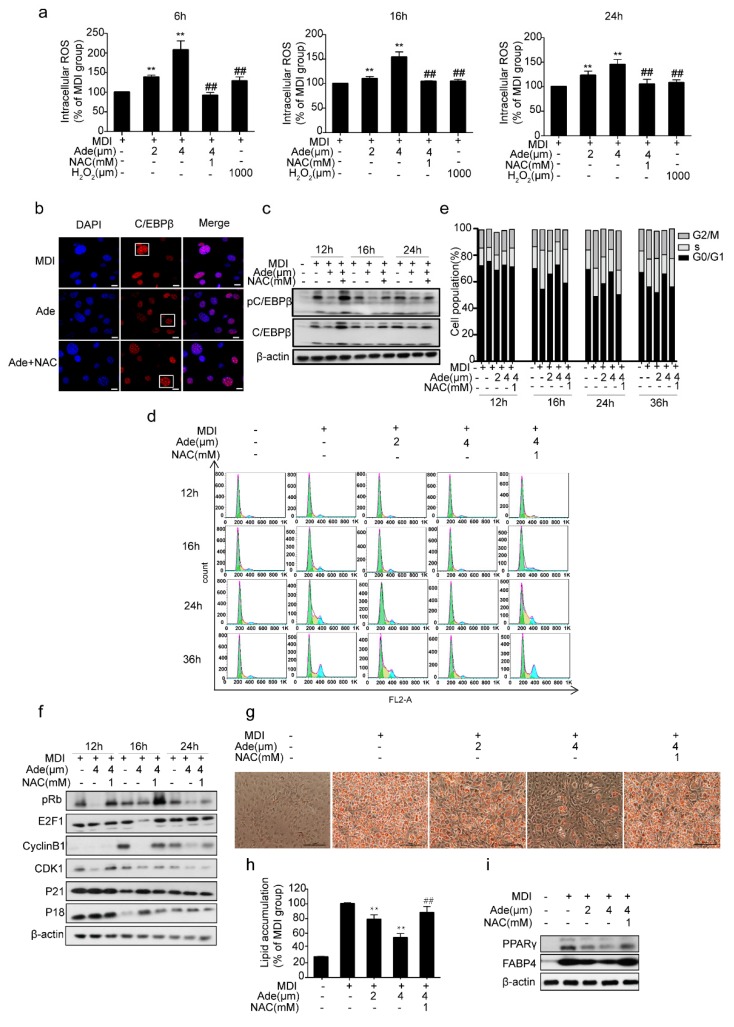
Adenanthin inhibited 3T3-L1 adipogenesis by regulating ROS. (**a**) Intracellular ROS levels of 3T3-L1 cells treated with MDI, and/or adenanthin (2, 4 μM), NAC (1 mM), H_2_O_2_ (1000 μM) for 6 h, 16 h and 24 h during MCE. Date are presented as mean ± SD of three independent experiments; ** *p* < 0.01 versus MDI group, ## *p* < 0.01 versus 4 μM of adenanthin group. (**b**) Representative images of immunostained-C/EBPβ in 3T3-L1 cells after inducing to differentiation for 16 h, scale bars, 10 μM. (**c**) Protein levels of pC/EBPβ and C/EBPβ of 3T3-L1 cells treated with MDI, and/or adenanthin (4 μM), NAC (1 mM) for 12 h, 16 h, and 24 h during MCE. (**d**) Flow cytometry analysis of 3T3-L1 cells treated with MDI, and/or adenanthin (2, 4 μM), NAC (1 mM) for 12, 16, 24, or 36 h. (**e**) Cell cycle Quantification of the cells from d. Date are averages of three independent experiments. (**f**) Protein expressions of pRB, E2F1, CylinB1, CDK1, P21, and P18 by western blotting of 3T3-L1 cells treated with MDI, and/or adenanthin (4 μM), NAC (1 mM) for 12 h, 16 h, or 24 h. (**g**) Representative images of 3T3-L1 adipocytes treated with MDI, and/or adenanthin (2, 4 μM), NAC (1 mM) were stained by Oil Red O. Scale bars, 100 μM. (**h**) Quantification of lipid accumulation by measuring Oil Red O intensity of 3T3-L1 cells from (**g**). Date are presented as mean ± SD of three independent experiments; ** *p* < 0.01 versus MDI group, ## *p* < 0.01 versus the 4 μM adenanthin group. (**i**) Protein expressions of PPARγ and FABP4 of 3T3-L1 cells from (**g**).

Several studies have previously reported that adenanthin treatment induced a moderate increase in intracellular ROS in MCE [28,30,32]. To explore the mechanism by which adenanthin affects 3T3-L1 cell differentiation, we estimated the intracellular ROS levels after treatments with adenanthin during MCE of 3T3-L1 differentiation. Surprisingly, 2 μM adenanthin-treated cells after 6, 16, and 24 h did increase intracellular ROS production approximately by 37%, 10%, and 23%, respectively. Furthermore, incubation of cells with 4 μM adenanthin increased intracellular ROS production even higher than that of 2 μM adenanthin (to 108%, 54%, and 45% after 6, 16, or 24 h), and the level of ROS by 4 μM adenanthin was even greater than the intracellular ROS level induced by exogenously supplemented with 1000 μM H_2_O_2_ in culture medium (Figure 4a). Moreover, incubation of cells with 4 μM adenanthin and 1 mM NAC (H_2_O_2_ cleaner) rescued ROS level back to that of the control (Figure 4a), supporting that adenanthin markedly enhanced intracellular ROS level during MCE of adipocyte differentiation.

Because C/EBPβ is required for initiating MCE and for activating expressions of transcription factors C/EBPα and PPARγ [16], it is considered as one of the crucial transcription factors expressed during MCE [33]. Even the expression of C/EBPβ is activated soon after the induction of differentiation, it does not bind to DNA until cells enter the S phase. Once C/EBPβ binds to DNA, it becomes localized to centromeres and results in a characteristic “punctate” pattern (Figure 4b, lane1, column2) [20]. Previous data have shown that the DNA binding activity of C/EBPβ is altered in an oxidative environment [22,34], and consistent with the reports, the immunofluorescence staining of C/EBPβ became diffuse when the cells were treated with adenanthin for 16 h (Figure 4b, lane2, column2). The co-treatment of cells with adenanthin and NAC converted the diffused pattern of C/EBPβ to punctate pattern (Figure 4b, lane3, column2). In addition, C/EBPβ acquires its DNA binding activity until it is phosphorylated by GSK3β [35], we then measured the effect of adenanthin on the expression and phosphorylation of C/EBPβ of the cells. We observed that adenanthin markedly reduced the expression and phosphorylation of C/EBPβ, whereas the combination of adenanthin with NAC recovered the expression and phosphorylation of C/EBPβ (Figure 4c). Therefore, adenanthin reduced C/EBPβ transcriptional activity by inhibiting the expression and characteristic DNA binding of C/EBPβ during MCE because of the increased intracellular ROS production.

Furthermore, we added NAC with adenanthin together for recovering the ROS level back to the level of control (Figure 4a) and monitored the changes in DNA content of 3T3-L1 cells. The result showed that the percentage of cells in the G0/G1 phase of the group treated with adenanthin and NAC was recovered to the normal level, strongly supporting the idea that adenanthin enhanced ROS production and then delayed MCE (Figure 4d,e).

Moreover, we examined the expression of the cell cycle regulators which control the G1/S transition. As shown in Figure 4f, the phosphorylation of RB and expression of E2F1 were markedly decreased after treatment with adenanthin during MCE. In contrast, the combination of adenanthin and NAC increased the levels of the proteins. Moreover, adenanthin induced the downregulation of its upstream regulators of RB-E2F1, including Cyclin B1 and Cdk1, but modestly increased protein levels of P21 and P18 during the first 24 h of the cell cycle, whereas the combination of adenanthin with NAC recovered the levels of these proteins. Together, these data revealed that adenanthin caused a G0/G1 cell cycle arrest by inhibiting the RB-E2F1 pathway mostly because of the increased intracellular ROS production.

Finally, adenanthin treatment inhibited the differentiation of 3T3-L1 cells, whereas the combination of adenanthin with NAC induced cells to differentiate normally (Figure 4g). Quantification of Oil Red O intensity showed that treatments with 2 or 4 μM adenanthin or with 4 μM adenanthin and 1 mM NAC decreased the lipid content by 21%, 46%, and 12%, respectively (Figure 4h). In addition, adenanthin significantly reduced the levels of the two key differentiation regulators PPARγ and FABP4, whereas the protein levels were recovered after adding NAC, supporting that the adenanthin-induced inhibition of adipogenesis is the result of increased intracellular ROS level (Figure 4i).

### 2.5. Adenanthin Delayed the HFD-Induced Development of Obesity

**Figure 5:**
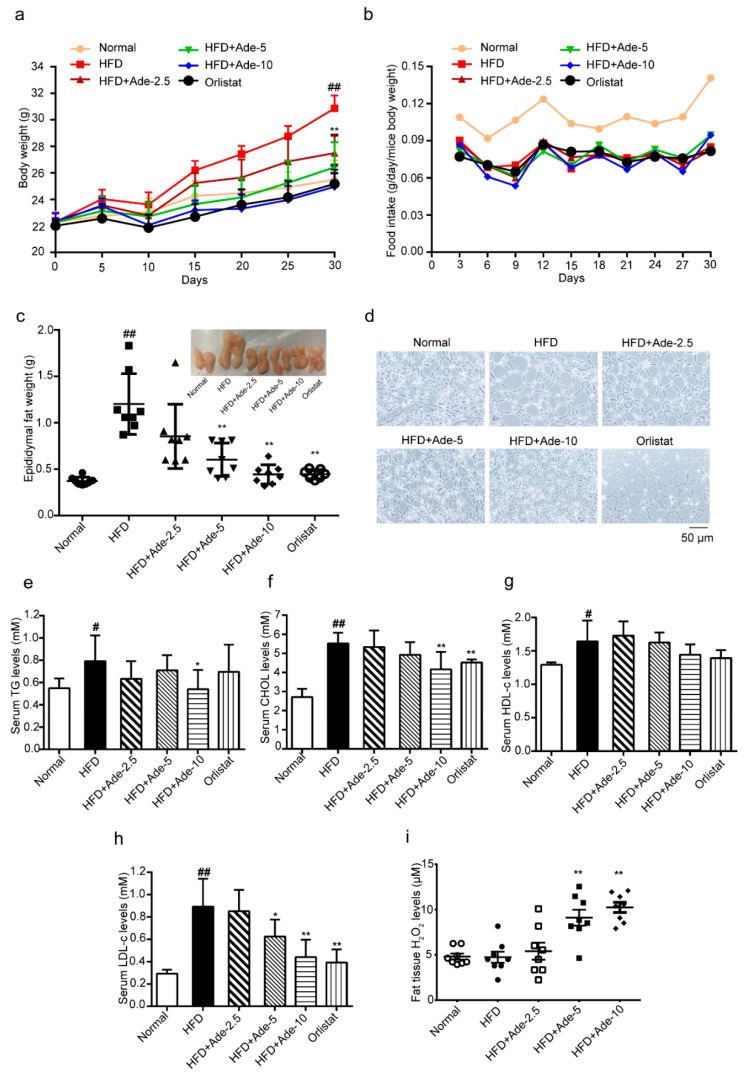
Adenanthin inhibited the development of obesity in high-fat diet (HFD) induced mice. (**a**) The time-lapse of body weight during 30-day treatments of mice by normal diet or HFD with adenanthin (2.5, 5, or 10 mg/kg/day) or orlistat (60 mg/kg/day). (**b**) Food intake during the inducing of obesity of mice from (**a**). (**c**) Weights of epididymal fat tissue of mice from (**a**). (**d**) Representative H&E staining images of epididymal adipose tissue from (**a**,**c**). Scale bars, 50 μM. (**e**–**h**) Serum TG, CHOL, HDL-c, and LDL-c levels of mice from (**a**). (**i**) The H_2_O_2_ level of epididymal adipose tissue from mice (**a**,**c**). Date are presented as mean ± SD; * *p* < 0.05, ** *p* < 0.01 versus HFD-induced group; # *p* < 0.05, ## *p* < 0.01 versus normal diet group, *n* = 8.

Next, we investigated the effect of adenanthin during HFD-induced development of obesity. Mice were fed with a HFD supplemented diet with or without adenanthin (2.5, 5 or 10 mg/kg/day) for 10 ten continuous courses during the early stage of induction of obesity (3-week old). Each course included a continuous 2-day injection and 1-day break [30]. After 30 days, the HFD-fed group showed obviously increased body weight compared to the normal diet fed group, whereas supplementation of HFD with adenanthin (2.5, 5, or 10 mg/kg/day) or orlistat (60 mg/kg/day) significantly reduced both the whole-body weight and accumulated gain of body weight (Figure 5a and Table 1). During the period of HFD inducing, there were no significant differences in food intake among the adenanthin, orlistat, and HFD group (Figure 5b). Furthermore, adenanthin or orlistat treatments dramatically reduced epididymal fat weights (Figure 5c). In addition, the average adipocyte size in epididymal adipose tissue was significantly smaller in adenanthin-treated mice (Figure 5d and Supplementary Figure S2). Moreover, the lipid metabolic variables in the serum of mice, serum triglycerides (TG), cholesterol (CHOL), and low-density lipoprotein cholesterol (LDL-c) levels in adenanthin-treated mice were shown tendency being lower than those of control mice, except the high-density lipoprotein cholesterol (HDL-c) level was not changed (Figure 5e–h). Finally, the level of H_2_O_2_ in epididymal adipose tissue of adenanthin-treated mice was significantly higher than that of control mice (Figure 5i), which is totally consistent with the result of 3T3-L1 in vitro.

## 3. Discussion

In this work, we showed that adenanthin functioned its anti-adipogenic effect by increasing the intracellular ROS level during MCE and then affected two processes: (1) inhibited the RB-E2F1 signaling pathway and results in a G0/G1 cell cycle arrest during MCE at the early stage of adipocyte differentiation; (2) reduced C/EBPβ transcriptional activity by inhibiting the expression and activity of C/EBPβ during MCE. These results were in line with the inhibitory effect of adenanthin on HFD-induced development of obesity in mice.

ROS, oxygen-derived small molecules, react readily with various chemical structures including proteins, lipids, sugars, and nucleic acids [36]. In recent years, there has been an accumulating understanding of ROS as signaling molecules [37]. Most research groups now believe that a regulated basal level of ROS is necessary and advantageous for maintenance of cell functions, such as proliferation, differentiation, and survival [38,39]. Increasing evidence has indicated that ROS plays a key role for adipogenesis, but whether the increased ROS is positive or negative for adipogenesis is still controversial. Some studies demonstrate that ROS promotes adipocytes to differentiate [22,23], however, other studies show that ROS is indicated to inhibit adipocyte differentiation by reducing the DNA-binding activity of C/EBPβ [24] and suppressing MCE [25]. Consistent with these findings [24,25], in our case, increased ROS inhibited adipogenesis, which was rescued by treatment of cells with adenanthin supplemented with H_2_O_2_ cleaner NAC (Figure 4). Furthermore, we exogenously treated cells with various doses of H_2_O_2_ (50 to 1200 μM) and found that lower doses (100, 200, and 400 μM) of H_2_O_2_ promoted 3T3-L1 differentiation, whereas higher doses (800, 1000, and 1200 μM) of H_2_O_2_ inhibited 3T3-L1 differentiation (Supplementary Figure S1a–c), implying that “positive” or “negative” roles of H_2_O_2_ relies on its level as previous report [26]. 

It is well known that adipogenesis occurs in several stages and involves a cascade of transcription factors [40]. Our results showed that adenanthin functions in MCE (Figure 3 and Figure 4). When it was induced to differentiation, growth-arrested 3T3-L1 preadipocytes synchronously re-enter the cell cycle and undergo MCE followed by expression of genes that produce the adipocyte phenotype. MCE is a prerequisite for differentiation of 3T3-L1 preadipocytes into adipocytes [16]. Therefore, cell cycle regulation at the early stage of adipogenesis has been considered as a strategy for modulating adipogenesis [41]. Recently, many natural products have been reported to be anti-obesity agents that can inhibit MCE of 3T3-L1 adipocytes [41,42,43]. In our work, we found that adenanthin could attenuate cell cycle progression through a G0/G1 arrest during MCE by decreasing protein levels of the phosphorylation of RB, E2F1, Cyclin B1, and Cdk1 and modestly increased protein levels of P21 and P18 (Figure 3 and Figure 4d–f). As the cells cross the G1/S checkpoint, C/EBPβ acquires DNA-binding activity. Coincident with the acquisition of DNA-binding activity, C/EBPβ binds to centromeres through consensus C/EBP-binding sites in centromeric satellite DNA [15,20]. Consistent with a previous study [43], our results showed that adenanthin inhibits the expression and activity of C/EBPβ during MCE (Figure 4b,c).

Finally, our results demonstrated that adenanthin reduced the growing body weight and adipose tissue mass during the early stage of induction of obesity (Figure 5a–d). Moreover, to evaluate the safety of adenanthin, the serum of HFD fed mice after a 30-day exposure to adenanthin was collected and serum aspartate amino transferase (AST), alamine amino transferase (ALT), and creatinine levels between vehicle and adenanthin-treated mice were evaluated, and the results were not significantly changed (Supplementary Table S1). Whether adenanthin is active during the late stage of obesity or has an anti-obesity effect is still unknown. In the future, we can monitor the activity of adenanthin during different stages of HFD-induced obesity. 

In summary, adenanthin, a natural *ent*-kaurane diterpenoid isolated from the herb *Isodon adenantha* in 1987 [27], was able to inhibit adipogenesis of 3T3-L1 and mouse embryonic fibroblasts (MEFs) through regulation of ROS and significantly reduced the growing body weight and adipose tissue mass during HFD-induced development of obesity in mice, revealing adenanthin may be a potential agent for prevention of obesity. 

## 4. Materials and Methods 

### 4.1. Reagents 

High-glucose Dulbecco’s modified Eagle’s medium (DMEM), newborn calf serum (CS), and fetal bovine serum (FBS) were purchased from Biological Industries (Cromwell, CT, USA). Dimethyl sulfoxide (DMSO), 3-isobutyl-1-methylxanthine (IBMX), hydrogen peroxide solution (H_2_O_2_) and Oil Red O were purchased from Sigma-Aldrich (St. Louis, MO, USA). Insulin was purchased from Roche (Basel, Switzerland) and dexamethasone (DEX) was purchased from Adamas (Zürich, Switzerland). Propidium iodide (PI) and reactive oxygen species assay kit (DCFH-DA) were purchased from Beyotime (Shanghai, China). *N*-acetyl-l-cysteine (NAC) was purchased from Meilunbio (Dalian, China). The antibodies against FABP4, PPARγ, pRb, CDK1, and CyclinB1 were purchased from Cell Signaling Technology (Beverly, MA, USA), the antibodies against E2F1, P21, P18, phospho-C/EBPβ (T235 + T188), C/EBPβ was purchased from Abcam (Cambridge, MA, USA) and the β-actin antibody was purchased from Sigma-Aldrich (St. Louis, MO, USA).

### 4.2. Extraction and Isolation of Adenanthin 

Adenanthin was isolated from the dried aerial parts of *Isodon adenantha* (Diels) Hara. The air-dried and powdered aerial parts of *I. adenantha* (1.8 kg) were extracted three times with ethanol solution (3 × 7 L, each 3 days) at room temperature and filtered. The filtrate was dried and partitioned with EtOAc (3 × 4 L). The EtOAc partition (110 g) was applied to silica gel (200–300 mesh) column chromatography, eluting with CH_2_Cl_2_–Me_2_CO (1:0–0:1 gradient system), to give six fractions, which were decolorized on MCI gel, eluted with 90% MeOH–H_2_O, to yield fractions A–F. Fraction B (250 g), brown gum, was subjected to column chromatography over a silica gel (200–300 mesh) column, eluted with Petroleum ether–Me_2_CO (1:0–0:1) gradient system, to obtain five fractions, B1–B5. Adenanthin (16.5 g) was precipitated from fraction B2, it had a degree of purity >98% [27]. The NMR spectra, MS spectra, and HRESIMS spectra have been shown in supplementary data.

### 4.3. 3T3-L1 Culture and Differentiation

3T3-L1 mouse preadipocytes were purchased from the American Type Culture Collection (ATCC, Manassas, VA, USA). Cells were cultured in DMEM supplemented with 10% CS. Two days after confluence (day 0), cells were induced for differentiation with DMEM supplemented with 10% FBS, IBMX, DEX and insulin (designated hereafter as MDI) as previously described [44]. On day 3, the medium was changed to medium containing 10% FBS and 1 μg/mL insulin for 2 days (day 5), and then insulin was removed from 10% FBS-DMEM for another 2 days. The cells were fully differentiated into mature adipocytes on day 7.

### 4.4. Isolation, Culture and Differentiation of MEFs

MEFs were isolated from 13.5-day old embryos of C57BL/6 mice (purchased from Vital River Laboratory Animal Technology Co. Ltd., Beijing, China) as previously described [45]. The cells were maintained in DMEM containing 10% FBS at 37 °C in 5% CO_2_ atmosphere. For differentiation, 2 days after confluence (day 0), cells were placed in 10% FBS-DMEM containing 1 μM DEX, 500 μM IBMX and 1 μg/mL insulin. On day 4, the medium was changed to 10% FBS-DMEM containing 1μg/mL insulin for 2 days, and then replaced with 10% FBS-DMEM for 2 days. The cells were fully differentiated into mature adipocytes on day 8. 

### 4.5. Cell Viability Assay 

Cell viability was measured by the cell proliferation MTS kit (Promega Corporation, Madison, MI, USA) according to the manufacturer’s protocols. Briefly, the 3T3-L1 adipocytes were treated with various concentrations of adenanthin in 96-well plates for 7 days. The medium was removed, and the MTS solution was added to each well for 2 h. The absorbance was measured at 492 nm by using a microplate reader (Perkin Elmer Envision Multilabel reader). 

### 4.6. Oil Red O Staining

After removing the culture medium, 3T3-L1 and MEF cells were washed three times with PBS and subsequently fixed in 10% formaldehyde for 1 h at room temperature. After fixing, the cells were washed with water twice and one time with 60% isopropanol then the cells were stained with Oil Red O working solution for 30 min, washed with water, and then photos were taken under microscopy. To quantify the lipid accumulation, the stained 3T3-L1 and MEF cells were washed with 100% isopropanol and the absorbance was measured at 492 nm by using a microplate reader (Perkin Elmer Envision Multilabel reader). The absorbance in MDI group was standardized to 100%.

### 4.7. Cell Cycle Analysis

3T3-L1 cells were induced to differentiate in DMI medium with or without adenanthin and NAC for 12 h, 16 h, and 24 h. The cells were then harvested, washed with PBS, fixed in 75% cold ethanol overnight, washed with PBS, resuspended in 500 µL of PBS containing 100 µg/mL RNase for 30 min at 37 °C and subsequently incubated with the nuclear stain PI at a final concentration of 40 µg/mL for 15 min. The stained cells were analyzed by using a BD AccuriC6 flow cytometer (BD Biosciences, San Jose, CA, USA) and the cell cycle distribution was analyzed by using FlowJo software.

### 4.8. Western Blotting Analysis

Cells were lysed in RIPA extraction buffer (Beyotime, Haimen, China) on ice for 30 min, then protein samples were separated using 10% SDS-PAGE and transferred to a polyvinylidene fluoride membrane. The membranes were probed with primary antibodies specific for FABP4, PPARγ, pRb, CDK1, CyclinB1, E2F1, P21, P18, phospho-C/EBPβ (T235 + T188), C/EBPβ, and β-actin, followed by incubating with secondary antibodies and then developed using Western Lightning Chemiluminescence Reagent (Perkin-Elmer Life Science, MA, USA). 

### 4.9. Intracellular ROS Measurement

3T3-L1 cells were seeded in 12-well plates at 0.5 × 10^5^ cells/well and were induced to differentiate in the different treated conditions. The cells were then washed twice with DMEM and incubated with 10 μM DCFH-DA (Beyotime, Haimen, China) in DMEM for 20 min at 37 °C Intracellular fluorescence was detected using a BD AccuriC6 flow cytometer (BD Biosciences, CA, USA). The analysis of intracellular ROS level was performed using FlowJo software.

### 4.10. Immunofluorescence Staining

3T3-L1 cells were seeded on glass coverslips in 35-mm dishes. After two-day post-confluence, cells were induced to differentiate in various conditions for 16 h. The cells were washed with PBS and fixed with 4% formaldehyde for 10 min at room temperature, permeabilized with 0.1% TritonX-100 for 15 min. After being incubated with primary antibodies against C/EBPβ (Abcam, MA, USA) for 30 min at 37 °C, washed with PBS again and then incubated with an Alexa Fluor 594-conjugated anti-mouse secondary antibody (Jacksonimmuno, PA, USA) for 30 min at 37 °C. For DNA staining, cells were treated with 1 μg/mL 4′,6-diamidino-2-phenylindole for 10 min at room temperature and then washed with PBS. Images were taken using a confocal microscope (Olympus, Japan) and analyzed using FV10-ASW 2.1 Viewer software.

### 4.11. Animal Study

Three-week old male C57BL/6J mice were purchased from Vital River Laboratory Animal Technology Co., Ltd. (Beijing, China). They were housed and treated according to the guidelines of the Institutional Animal Care and Use Committee. As previously described [46], we fed the mice in sterile facilities with a 12 h/12 h light-dark cycle at a controlled temperature of 24 °C. After feeding with regular diet for 1 week, they were stratified randomized to six groups (*n* = 8/group) according to the weights. The six groups included a normal dietary group (10% calories from fat, Research Diets Inc.), a high-fat diet (HFD) fed group (60% calories from fat, HFD, Research Diets Inc.), three HFD groups with a series of adenanthin doses (2.5, 5 or 10 mg/kg/day) and a HFD group with orlistat treatment (60 mg/kg/day). Mice were administered with vehicle and adenanthin intraperitoneally (i.p.) or orlistat intragastrically (i.g.) for ten continuous courses. Each course included continuous 2-day injection and 1-day break [30]. 

During the process, body weight and food intake were monitored at indicated times points. Epididymal fat mass from mice were divided into two sets. One set were fixed in 4% paraformaldehyde overnight, dehydrated through ethanol gradients, infiltrated in xylene and then embedded in paraffin for hematoxylin and eosin (H&E) staining as previously described [46]. The other set of epididymal fat samples were stored at −80 °C for other analysis. The levels of triglyceride, cholesterol, low-density lipoprotein cholesterol, high-density lipoprotein cholesterol, creatinine, aspartate amino transferase and alamine amino transferase in serum were measured using the commercial kits (Nanjing Jiancheng Bioengineering Institute, China). The level of H_2_O_2_ in epididymal adipose tissue was measured by the hydrogen peroxide assay kit (Beyotime, China) according to the manufacturer’s protocols. 

### 4.12. Statistical Analysis

The results are shown as means ± SD unless otherwise stated. Student’s *t*-test and one-way analysis of variance (ANOVA) were used to determine statistically significant differences between the two groups and in multiple-group comparisons respectively. Significance was determined as *p* < 0.05.

## 5. Patents

China patent, licensed, licensee: CN104292103B, a compound inhibits adipogenesis in 3T3-L1 preadipocytes and its application.

## Figures and Tables

**Table 1:** Adenanthin reduced body weight gain in mice.

	Initial Weight (g)	Final Weight (g)	Weight Gain (g)
Normal	22.29 ± 0.28	25.49 ± 0.51	3.20 ± 0.70
HFD (2% DMSO)	22.29 ± 0.23	30.86 ± 0.35 ^##^	8.58 ± 0.43 ^##^
HFD + Ade (2.5 mg/kg)	22.26 ± 0.12	27.50 ± 0.49 **	5.24 ± 0.47 **
HFD + Ade (5 mg/kg)	22.28 ± 0.24	26.43 ± 0.67 **	4.15 ± 0.57 **
HFD + Ade (10 mg/kg)	22.38 ± 0.22	24.95 ± 0.36 **	2.58 ± 0.49 **
HFD + Orlistat (60 mg/kg)	22.19 ± 0.17	25.17 ± 0.41 **	2.98 ± 0.28 **

Mice were fed for 30 days as described in the experimental section. Date are presented as mean ± SE; ** *p* < 0.01 versus HFD-induced group; ^##^ *p* < 0.01 versus normal diet group, *n* = 8. Normal: normal diet; HFD: high-fat diet; Ade: adenanthin.

## Supplementary Materials

The following are available online at http://www.mdpi.com/1420-3049/24/1/158/s1, The NMR spectra, MS spectra, and HRESIMS spectra of Adenanthin, Table S1: Effect of Adenanthin on Serum ALT, AST and Creatinine in Mice, Figure S1: Effect of H_2_O_2_ on 3T3-L1 Adipogenesis. Figure S2: The epididymal fat adipocyte size quantification of cells from Figure 5d.
