# Supplementary material for: Adenanthin, a Natural ent-Kaurane Diterpenoid Isolated from the Herb Isodon adenantha Inhibits Adipogenesis and the Development of Obesity by Regulation of ROS
Source: Molecules. 2019 Jan 3;24(1):158. doi: 10.3390/molecules24010158 (PMC6337096; doi:10.3390/molecules24010158)
Supplement: Supplementary file 1 [file molecules-24-00158-s001.pdf]

Adenanthin, a natural *ent-kaurane* diterpenoid isolated from the herb *Isodon adenantha* inhibits adipogenesis and the development of obesity by regulation of ROS

Jing Hu [1,2,3], Xing-Ren Li [1,2], Wei-Feng Tian [1,2#], Yan-Ting Lu [1,2], Yu-Hui Xu [1,2,3], Fang Wang [1,2,3], Wan-Ying Qin [1,2], Xiu-Li Ma [1,2], Pema-Tenzin Punuo [1,3,*] and Wen-Yong Xiong [1,3,*]

[#] Current address: Kunming Institute of Zoology, Chinese Academy of Sciences, Kunming 650223, China;

Supporting data

1. The NMR spectra of adenanthin
2. The MS spectra of adenanthin
3. The HRESIMS spectra of adenanthin
4. Supplementary table1 (Table S1)
5. Supplementary figure1 (Figure S1)
6. Supplementary figure2 (Figure S2)

1. Copies of ^{1}H NMR spectra of Adenanthin

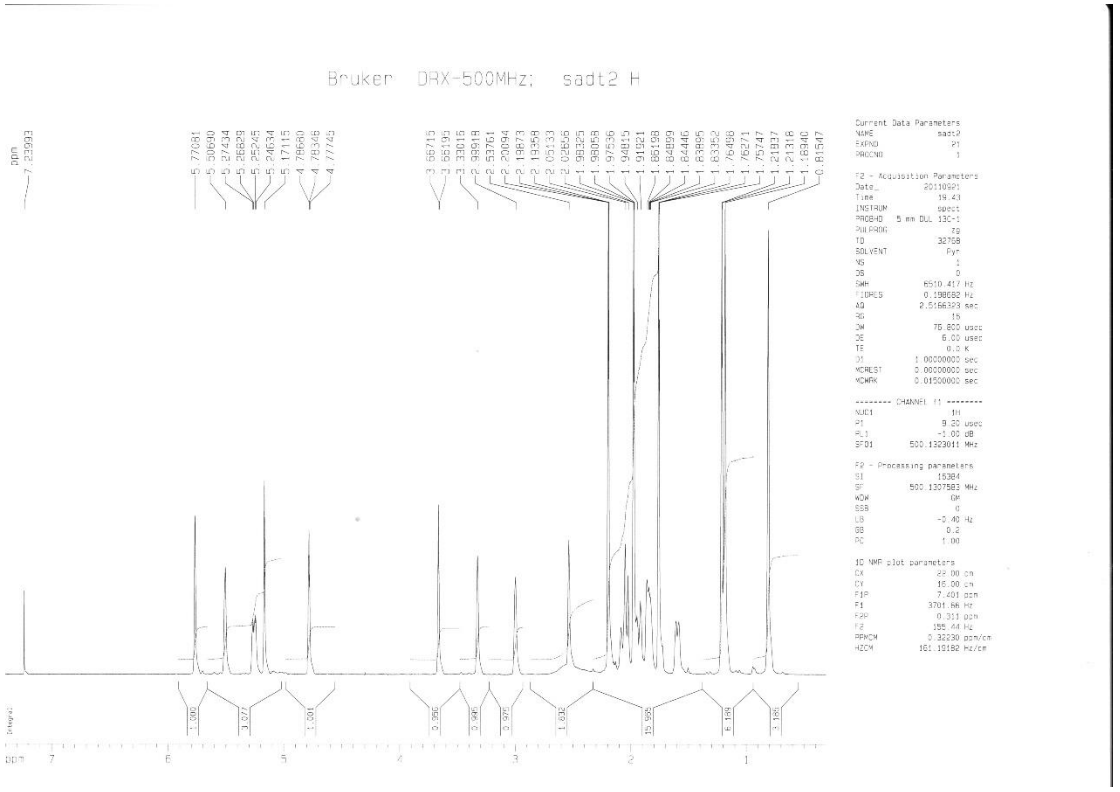

Copies of 13C NMR spectra of Adenanthin

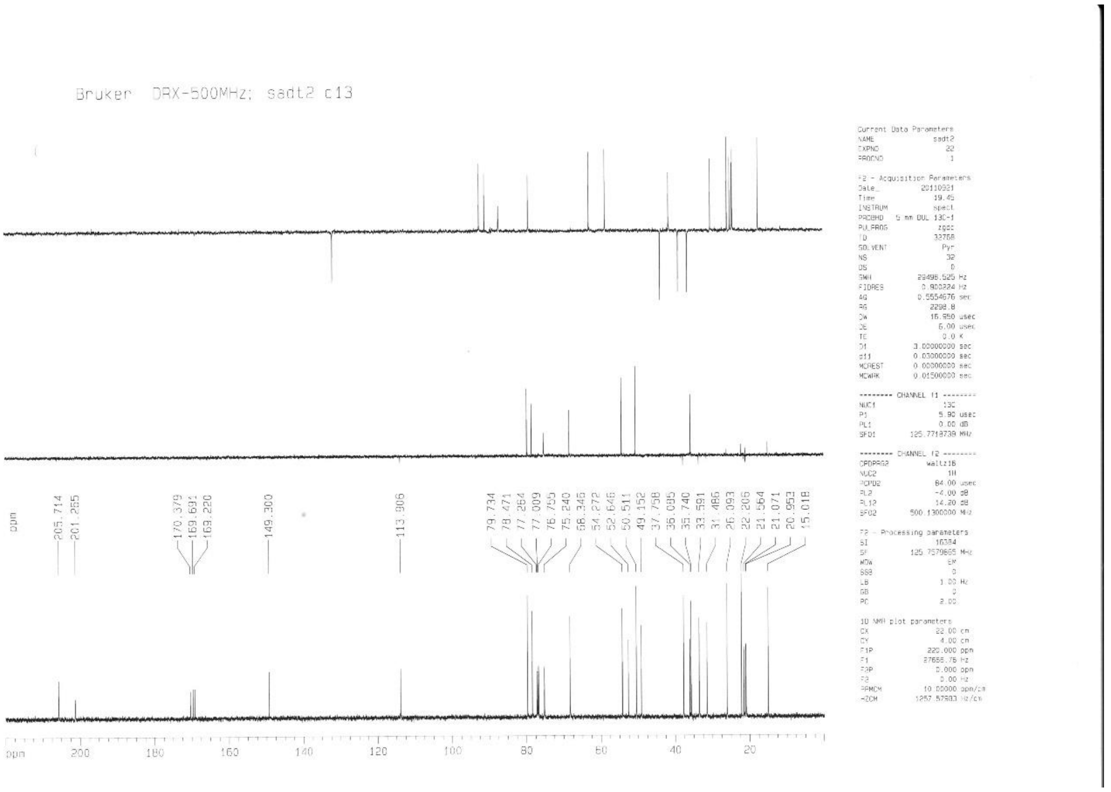

2. Copies of MS spectra of Adenanthin

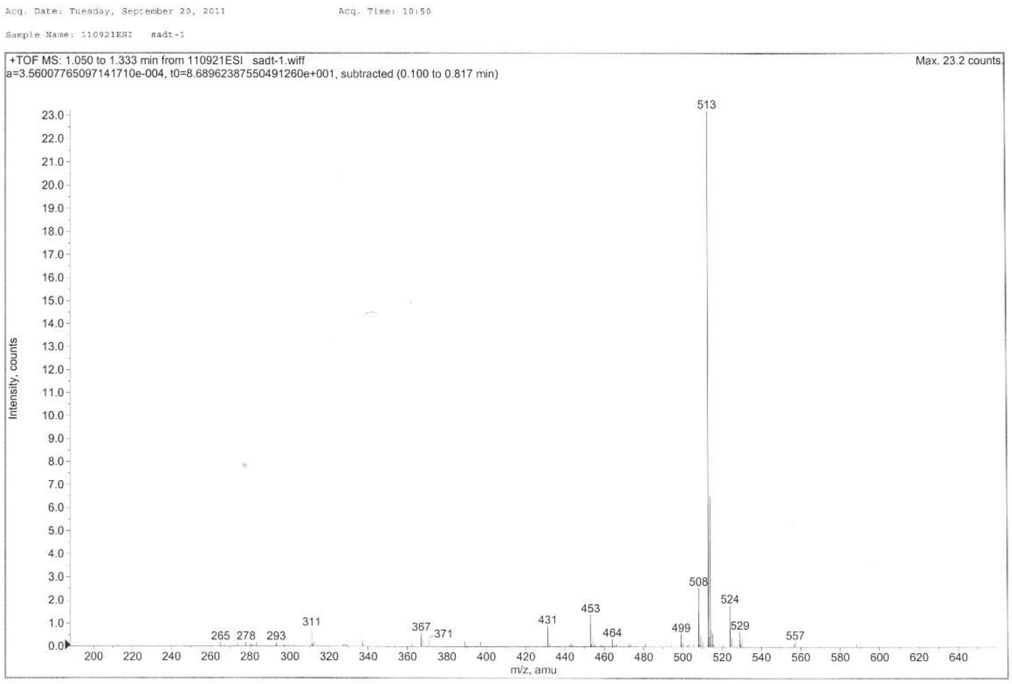

3. The HRESIMS spectra of adenanthin

Qualitative Analysis Report

**Data Filename**	sadt-1.d	**Sample Name**	sadt-1
**Sample Type**	Sample	**Position**	P1-A1
**Instrument Name**	Instrument 1	**User Name**	
**Acq Method**	s.m	**Acquired Time**	12/24/2018 11:55:22 AM
**IRM Calibration Status**	Success	**DA Method**	Default.m
**Comment**			

Sample Group **Info.**
Acquisition SW 6200 series TOF/6500 series
Version Q-TOF B.05.01 (B5125.2)

User Spectra

Fragmentor Voltage	Collision Energy	Ionization Mode
135	0	ESI

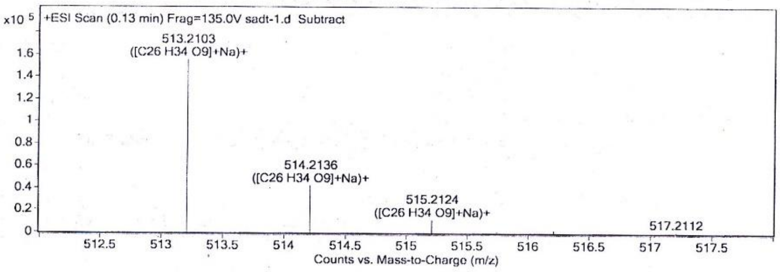

Peak List

m/z	z	Abund	Formula	Ion
508.2549	1	87311.02		
513.2103	1	155241.19	C26 H34 O9	(M+Na)+
514.2136	1	42244.24	C26 H34 O9	(M+Na)+
529.1846	1	142186.94		
530.1876	1	37896.65		
1003.4331	1	271884.41		
1004.4363	1	151853.25		
1005.4386	1	50421.89		
1019.4071	1	41739.49		
1020.4083	1	24297.93		

Formula Calculator Element Limits

Element	Min	Max
C	3	60
H	0	120
O	0	30

Formula Calculator Results

Formula	CalculatedMass	CalculatedMz	Mz	Diff. (mDa)	Diff. (ppm)	DBE
C26 H34 O9	490.2203	513.2095	513.2103	-0.80	-1.56	10.0000

--- End Of Report ---

Agilent Technologies Page 1 of 1 Printed at: 11:56 AM on: 12/24/2018

4. Supplementary table1 (Table S1)

Table S1. Effect of Adenanthin on Serum ALT、AST and Creatinine in Mice

Serum concentration	ALT U/L	AST U/L	Creatinine μM
Normal	8.87±1.23	14.86±1.49	81.73±1.89
HFD (2% DMSO)	13.15±1.74	13.45±1.30	88.57±4.29
HFD+Ade (2.5 mg/kg)	11.03±1.06	11.67±1.90	84.82±6.56
HFD+Ade (5 mg/kg)	14.71±2.86	16.05±1.36	78.17±2.07
HFD+Ade (10 mg/kg)	16.75±3.39	14.40±1.48	91.89±6.89
HFD+Orlistat (60 mg/kg)	15.76±2.79	20.46±2.14	80.87±3.91

Mice were treated with vehicle (2% DMSO), adenanthin (2.5, 5 or 10 mg/kg) or orlistat (60mg/kg) once a day for continuous ten courses. Each course included continuous 2- day injection and 1- day break. The mice were fasted 4 h before serum collected. Error bars represent SE, n=8. ALT: alamine amino transferase; AST: aspartate amino transferase; CK: creatine kinase; Normal: normal diet; HFD: high-fat diet; Ade: Adenanthin.

5. Supplementary figure1 (Figure S1)

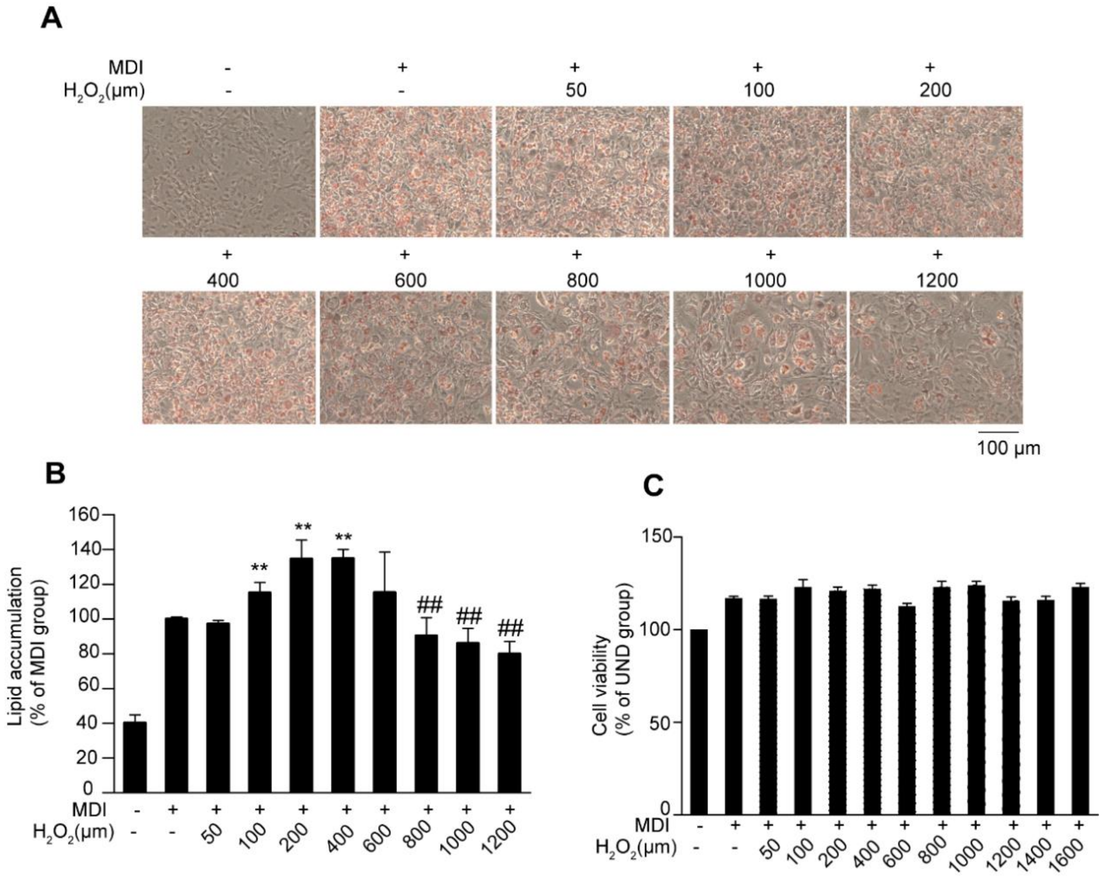

Figure S1. Effect of H2O2 on 3T3-L1 adipogenesis. (A) Lipid accumulation of 3T3-L1 adipocytes treated with various doses of H2O2 were visualized by Oil Red O staining, Scale bars, 100 μm. (B) Quantification of accumulated lipid content by measuring absorbance at 492 nm. (C) Effect of H2O2 on 3T3-L1 cell viability after 7 days of differentiation. Date are presented as mean ± SD of three independent experiments; **p<0.01 versus MDI group; ##p<0.01 versus MDI group.

6. Supplementary figure2 (Figure S2)

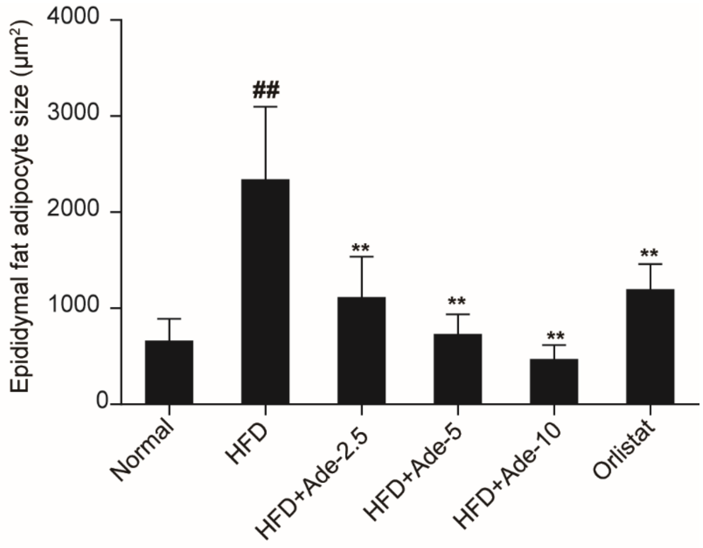

Figure S2. The epididymal fat adipocyte size quantification of cells from Figure 5d. The date are presented as mean ± SD; **p<0.01 versus HFD-induced group; ##p<0.01 versus normal diet group.
